# Design and Characterization of an HRC-Derived Peptide Inhibitor of Canine Coronavirus Spike-Mediated Fusion

**DOI:** 10.3390/pathogens15030315

**Published:** 2026-03-14

**Authors:** Valentina Iovane, Rosa Giugliano, Antonio Gentile, Roberta Della Marca, Laura Di Clemente, Annalisa Chianese, Serena Montagnaro, Anna De Filippis, Massimiliano Galdiero, Carla Zannella

**Affiliations:** 1 Department of Agricultural Sciences, University of Naples Federico II, 80055 Portici, Italy; valentina.iovane@unina.it (V.I.); gentileanto91@gmail.com (A.G.); 2 Department of Woman, Child and General and Specialized Surgery, University of Campania Luigi Vanvitelli, 80138 Naples, Italy; rosa.giugliano@unicampania.it (R.G.); roberta.dellamarca@unicampania.it (R.D.M.); laura.diclemente@unicampania.it (L.D.C.); annalisa.chianese@unicampania.it (A.C.); anna.defilippis@unicampania.it (A.D.F.); massimiliano.galdiero@unicampania.it (M.G.); 3 Department of Veterinary Medicine and Animal Production, University of Naples Federico II, 80137 Naples, Italy; serena.montagnaro@unina.it; 4 Department of Life Sciences, Health and Health Professions, Link Campus University, 00165 Rome, Italy; 5 UOC Virology and Microbiology, University Hospital “Luigi Vanvitelli”, 80138 Naples, Italy

**Keywords:** CCoV, heptad repeats, fusion assay, viral inhibition, spike protein, docking

## Abstract

Canine coronavirus (CCoV), an alphacoronavirus belonging to the *Coronaviridae* family, is primarily associated with enteric infections in dogs. The ongoing evolution of coronaviruses through genetic recombination and mutation leads to the emergence of novel strains with increased pathogenicity, thereby raising the risk of cross-species transmission and spillover events. In this context, viral entry inhibitors represent a promising strategy, as they can serve as pivotal tools to prevent initial infection and subsequent viral replication. The S2 subunit of the spike (S) glycoprotein contains two heptad repeat regions (HRN and HRC), which play essential roles in the conformational changes required for viral fusion. In this study, we describe the design, synthesis, and functional evaluation of a peptide derived from the HRC domain of the CCoV S glycoprotein. First, we assessed the cytotoxicity of the CCoV-HRC peptide in two cell lines, HE293T and A72, and determined CC_50_ values > 100 μM. At non-toxic concentrations, the peptide effectively blocked membrane fusion mediated by the CCoV S glycoprotein and significantly reduced viral infection, as demonstrated both in cell–cell fusion assays and in live virus experiments. These findings were supported by in silico docking and molecular dynamics simulations, which provided structural insight into the interaction between CCoV-HRC and the S fusion core. Then, molecular analyses were conducted to evaluate the expression of the gene encoding the viral S protein, confirming the antiviral potential of CCoV-HRC peptide. Overall, these findings provide a solid foundation for the development of peptide-based therapeutics to treat or prevent CCoV infections.

## 1. Introduction

Coronaviruses (CoVs) are enveloped, single-stranded, positive-sense RNA viruses capable of infecting a wide range of hosts, including humans, mammals, and birds [1]. These viruses can cause respiratory, hepatic, gastrointestinal, and neurological diseases of varying severity. Based on phylogenetic analysis, CCoVs are divided into four genera, *Alphacoronavirus* and *Betacoronavirus*, which primarily infect mammals, and *Gammacoronavirus* and *Deltacoronavirus*, which mainly infect birds but can occasionally infect mammals [2,3]. Canine enteric coronavirus (CCoV) belongs to the *Alphacoronavirus* genus and is typically associated with self-limiting enteritis in dogs. Two genotypes have been described, CCoV type I (CCoV-I) and CCoV type II (CCoV-II), which are distinguished by differences in their spike (S) proteins, sharing only 54% sequence identity [4]. While most CCoV infections are mild, certain strains can cause severe systemic disease [5]. For example, the pantropic strain CB/05 has been reported to induce fatal infections in dogs. Importantly, CCoV can also pose a potential risk for cross-species transmission [6]. In 2018, a novel recombinant canine–feline alphacoronavirus (genotype II) was isolated from a child hospitalized for pneumonia in Malaysia and was designated as CCoV (HuPn)-2018 [7]. A closely related strain was subsequently identified in a doctor who developed fever and malaise after traveling to Haiti [8], and very recently, Phan et al. detected CCoV-HuPn-2018 in several patients hospitalized for pneumonia in Vietnam [9].

At the genomic level, CCoV type II shares high similarity with other alphacoronaviruses, including feline CoV (FCoV) and porcine transmissible gastroenteritis virus (TGEV), highlighting its potential for cross-species transmission and emphasizing the importance of effective and broad antiviral interventions [10].

A critical factor determining CoV tissue tropism and host range is the ability of the S protein to bind to host cellular receptors. The S protein mediates viral attachment, folds as a homotrimer, and facilitates fusion with host cell membranes [11]. It is synthesized as a precursor that is subsequently cleaved into a surface subunit responsible for receptor binding (S1) and a transmembrane subunit responsible for membrane fusion (S2) [12]. In CCoV, the S protein binds the aminopeptidase N (APN) receptor on the target cell and mediates subsequent virus–cell fusion [13]. Upon receptor engagement, the fusion machinery undergoes a major conformational change, exposing a hydrophobic fusion peptide (FP) that inserts into the target membrane. The ectodomain of the S2 subunit contains two heptad repeats (HR) regions: an N-terminal HR domain (HR1) adjacent to the FP and a C-terminal HR region (HR2) proximal to the transmembrane anchor [14]. Structural studies of viral fusion proteins have shown that these HR regions assemble into a six-helix bundle (6HB), a key intermediate that drives membrane apposition and viral entry.

Peptides derived from HR regions can mimic components of the fusogenic structure and interfere with 6HB formation. A paradigmatic example is the human immunodeficiency virus type 1 (HIV-1) HR2-derived peptide T-20, which was licensed by the Food and Drug Administration (FDA) in 2003 and has been successfully incorporated into clinical regimens with strong antiviral efficacy [15,16]. The same strategy has proven effective against several CoVs, including severe acute respiratory syndrome coronavirus (SARS-CoV) and type 2 (SARS-CoV-2), Middle East respiratory syndrome (MERS-CoV), and feline infectious peritonitis virus (FIPV) [17,18,19], supporting the broad applicability of HR-derived fusion inhibitors.

Herein, to identify a potent inhibitor of CCoV entry, we designed a synthetic peptide, termed CCoV-HRC, overlapping the predicted HRC region of the CCoV S protein. This peptide could block 6HB formation and thereby inhibit viral membrane fusion and entry. To further elucidate the molecular basis of its antiviral activity, in silico docking and molecular dynamics simulations were performed to characterize the interaction between the peptide and the spike fusion core.

## 2. Materials and Methods

### 2.1. Plasmids and Peptides

The genes encoding the CCoV S protein and the canine APN receptor were codon-optimized, synthesized, and subcloned into the mammalian expression vector pCAGGS-neo. The following plasmids were purchased from Epoch Life Science (Missouri City, Texas, United States): pCAGGS- puro-APN canis-mVenus (GS77106-16), pCAGGS-puro-CCoV_S (GS77106-6), pCAGGS-mCherry Kan (GS590275), pCAGGS-puro-S-nCoV (GS66473-1), pCAGGS-puro-ACE2-Venus (GS66539), pCAGGS-Neo-Alpha-beta-gal (GS61265-2), and pCAGGS-Omega-beta-gal (GS61265-3).

The CCoV-HRC peptide was synthesized by Synpeptide Co. (Shanghai, China). After purification, liquid chromatography–mass spectrometry (LC-MS) analysis confirmed its identity and high purity (>95%), with the observed mass matching the theoretical value. As positive and negative controls, oreochromycin-1 and a scrambled version of the CCoV-HRC peptide (sequence: ENLLVLNNATLTFITEDNITIFLIRSENLNEHYLAENLTDVGLNKWID), maintaining the same amino acid composition but in a randomized order, were respectively synthesized and purified under the same conditions.

### 2.2. Cell Lines and Virus

Canine fibrosarcoma cells (A72; ATCC CRL-1542) and human embryonic kidney cells (HEK-293T; ATCC CRL-3216) were acquired from the American Type Culture Collection (ATCC; Manassas, VA, USA). Both cell lines were maintained in Dulbecco’s Modified Eagle Medium (DMEM) containing 4.5 g/L glucose (Microtech, Naples, Italy), supplemented with 10% fetal bovine serum (FBS; Microtech) and 100 IU/mL penicillin/streptomycin solution (Himedia, Naples, Italy). Cells were maintained at 37 °C in a humidified incubator with 5% CO_2_. CCoV type II strain S/378 was kindly provided by Prof. C. Buonavoglia (University of Bari Aldo Moro, Bari, Italy) and propagated on A72 cells.

### 2.3. Cell Proliferation Assay

A72 and HEK-293T cells were seeded in 96-well plates at an initial density of 2 × 10^4^ cells per well and incubated overnight at 37 °C. Serial dilutions of the CCoV-HRC peptide (3–100 µM) were added to the cells, which were then incubated for 24 hours (h) at 37 °C. Cell proliferation was assessed using the 3-(4,5-dimethyl-2-thiazolyl)-2,5-diphenyl-2H-tetrazolium bromide (MTT) assay (Sigma-Aldrich, St. Louis, MO, USA). Briefly, MTT was added to each well, and cells were incubated for 4 h at 37 °C. After incubation, the supernatant was discarded, and dimethyl sulfoxide (DMSO) was added to dissolve the formazan crystals [20]. Absorbance was measured at 570 nm using a microplate reader, and cell viability was calculated as follows:

$$cell\;viability\;(\%)=\frac{absorbance\;of\;treated\;cells}{absorbance\;of\;untreated\;cells}\;\times \;100$$

### 2.4. Fusion Assay

The fusion assay was performed using β-galactosidase (β-Gal) complementation in two populations of HEK-293T cells, as previously reported [21]. One cell population was transfected with the CCoV S protein, the β-gal α subunit, and the red fluorescent protein mCherry for visualization. The second population expressed the cellular receptor APN and the β-gal ω subunit. After 6 h of incubation at 37 °C, both cell populations were detached and overlaid to initiate fusion. The assay was conducted in the absence or presence of the CCoV HRC peptide at concentrations ranging from 3 to 50 μM. Fusion was initially assessed by observing cytopathic effects (CPEs) under fluorescence microscopy (ZOE Fluorescent Cell Imaging System; Bio-Rad, Hercules, CA, USA). Cell fusion was quantified by α-ω complementation. After cell lysis, the chemiluminescent substrate (Galacto-Star; Thermo Fisher Scientific, Waltham, MA, USA) was added, and luminescence was measured using a Tecan M1000PRO microplate reader (Tecan Trading AG, Männedorf, Switzerland).

$$fusion\;inhibition\;\left(\%\right)=1-\frac{absorbance\;of\;treated\;cells}{absorbance\;of\;untreated\;cells}\times 100$$

In addition to untreated controls, the scrambled peptide was tested at the same concentrations to confirm that inhibition was sequence-specific.

### 2.5. Viral Inhibition Assay

The antiviral activity of the CCoV-HRC peptide was evaluated using a tissue culture infective dose (TCID_50_) assay, as previously described [22]. Briefly, A72 cells were seeded in 96-well plates at a density of 2 × 10^4^ cells per well and incubated for 24 h. The virus (multiplicity of infection, MOI = 0.1) was pre-incubated with the peptide and then applied to the cells at a final MOI of 0.01. After viral adsorption, the inoculum was replaced with fresh culture medium, and cells were incubated for 48 h at 37 °C. Following incubation, cells were fixed with 4% formaldehyde and stained with 0.5% crystal violet to observe CPEs under a light microscope. Oreochromycin-1 (25 μM) and untreated infected cells were used as positive control (CTRL+) and negative control (CTRL−), respectively [23]. Similarly, the scrambled peptide served as a negative control to ensure that observed antiviral effects were due to the specific HRC sequence. The TCID_50_/mL was calculated using the Reed and Munch method, based on the dilution at which 50% of the wells exhibited CPEs [24].

### 2.6. Real-Time PCR

A72 cells were seeded in 24-well plates at a density of 1.4 × 10^5^ cells per well and incubated at 37 °C for 24 h. Cells were then infected as described in Section 2.5. After viral adsorption, fresh medium was added, and cells were incubated for 48 h at 37 °C.

Total RNA was extracted using TRIzol reagent (Thermo Fisher Scientific) and reverse-transcribed into cDNA with the 5× All-In-One RT MasterMix kit (Applied Biological Materials, Richmond, BC, Canada). The expression levels of the viral S protein gene were quantified by real-time quantitative PCR (qPCR) [25].

Cycle threshold (Ct) values of treated and infected cells were compared to those of infected and untreated controls and normalised to the housekeeping gene glyceraldehyde-3-phosphate dehydrogenase (GAPDH). Primer sequences were as follows: GAPDH (forward: 5′-CCTTTCATTGAGCTCCAT-3′; reverse: 5′-CGTACATGGGAGCGTC-3′); and CCoV S (forward: 5′-TTGAAGGTGCGCAGTTTAGC-3′; reverse: 5′-CAGTAACGCGGTCCATCAGT-3′). Relative quantification of gene expression was determined using the 2^−ΔΔCt^ method.

### 2.7. Statistical Analysis

Statistical analyses were conducted with GraphPad Prism version 9.5.1 for macOS (GraphPad Software, San Diego, CA, USA; www.graphpad.com). All experiments were carried out in triplicate and shown as mean ± standard deviation (SD). The CC_50_ (concentration causing 50% cytotoxicity) and IC_50_ (concentration causing 50% inhibition) values were determined using nonlinear regression analysis of dose–response curves. The selectivity index (SI), a key parameter for determining the therapeutic window, was calculated as the ratio of the CC_50_ to the IC_50_.

### 2.8. Homology Modelling and Docking Analysis

To investigate the interaction between the CCoV-HRC peptide and the spike fusion core, an in silico structural analysis was performed on CCoV strain S378, CCoV-HuPn-2018, and SARS-CoV-2. Viral protein sequences were retrieved from the NCBI database, and available crystallographic structures were obtained from the Protein Data Bank (PDB) (Table 1).

As no experimental structure was available for the CCoV strain S378, a three-dimensional model was generated by homology modelling using the SWISS-MODEL server [26]. Model quality was evaluated through QMEAN scoring, Ramachandran plot analysis, and MolProbity assessment. Structural refinement was subsequently performed using the Chiron server to minimize steric clashes and optimize backbone geometry [27,28]. The final model showed good stereochemical quality (global QMEAN score: 0.81 ± 0.05), with the majority of residues located in favored regions of the Ramachandran plot [29].

Protein–peptide docking was performed to evaluate the interaction between the modeled fusion cores and the CCoV-HRC peptide. Docking simulations were carried out using the LZerD web server without imposing interaction restraints. Generated complexes were clustered based on structural similarity and ranked according to the LZerD rank-sum score, which integrates van der Waals, electrostatic, and desolvation energy terms. The top-ranked complexes were selected for subsequent molecular dynamics simulations.

### 2.9. Molecular Dynamics Simulations

Molecular dynamics simulations (MDSs) were performed using GROMACS with the AMBER99SB-ILDN force field to investigate the stability and dynamic behavior of the spike fusion core–CCoV-HRC complexes. All systems were solvated in a cubic box with TIP3P water molecules and neutralized with counterions at physiological ionic strength (pH 7.2 conditions). Periodic boundary conditions were applied.

Energy minimization was conducted using the steepest descent algorithm to remove steric clashes, followed by equilibration phases under NVT and NPT ensembles. Production runs were then carried out for 10 ns at 310 K and 1 atm using a 2 fs time-step. Long-range electrostatic interactions were treated using the Particle Mesh Ewald (PME) method.

Trajectory analyses included root mean square deviation (RMSD) to evaluate structural stability, as well as hydrogen bond occupancy and electrostatic interaction profiling to assess complex stability over time. Binding free energy (ΔG) estimations were calculated from equilibrated trajectory frames. All the bioinformatics tools employed for molecular modelling, structural validation, molecular docking, and MDS are indicated in Table 2.

## 3. Results

### 3.1. Prediction of C-Terminal HR of CCoV

The HR2 region of the CCoV (strain S378, NCBI Gene Accession No KC175341) S glycoprotein was identified through structure-based multiple sequence alignment using the bioinformatics tool VectorBuilder.com, with known HR2 regions of coronaviruses, including a recent canine strain (CCoV-HuPn-2018, NCBI Gene Accession No QVL91811) and several other coronaviruses, such as SARS-CoV (NCBI Gene Accession No AAP13441), SARS-CoV-2 (NCBI Gene Accession No QHD43416.1), FIPV (NCBI Gene Accession No BAA06805), avian infectious bronchitis virus (IBV; NCBI Gene Accession No AAO34396), porcine epidemic diarrhea virus (PEDV; NCBI Gene Accession No KU558701), feline coronavirus (FCoV; NCBI Gene Accession No DQ848678), transmissible gastroenteritis coronavirus (TGEV; NCBI Gene Accession No AAB30949), and mouse hepatitis virus (MHV; NCBI Gene Accession No P11225). Based on this analysis, the following CCoV-HRC sequence was designed: DIFNATYLNLTGEINDLEFRSEKLHNTTVELAILIDNINNTLVNLEWL. Additionally, the percentage of identity and similarity, two important parameters for quantifying the relationship between two protein sequences, were calculated with respect to the HRC of other coronaviruses via VectorBuilder.com (Table 3).

In Table 3, the HRC sequences are organised by genus: α-CoVs, β-CoVs, and γ-CoVs. Based on the identity and similarity percentages, CCoV-HRC contains sequences that are highly conserved with CCoV-HuPn 2018 and moderately conserved with other alphacoronaviruses compared to other genera, indicating that any cross-inhibition phenomena based on this sequence may be more likely between closely related alphacoronaviruses than between different genera.

### 3.2. CCoV HRC Does Not Significantly Affect Cell Viability in HEK-293T and A72 Cells

**Figure 1:**
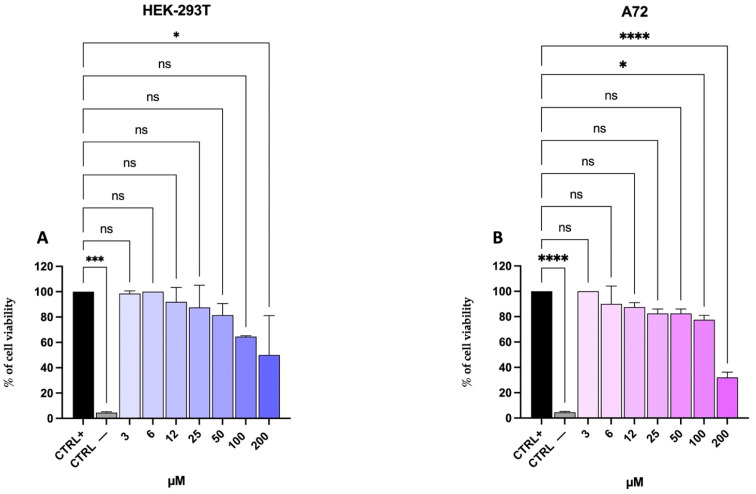
Evaluation of cell viability after 24 h of treatment with CCoV-HRC using the MTT assay. (**A**) Dose–response curve in HEK-293T cells. (**B**) Dose–response curve in A72 cells. The positive control (CTRL+) corresponds to untreated cells, whereas the negative control (CTRL−) corresponds to cells treated with 100% DMSO. Cell viability is expressed as a percentage relative to the positive control. Data are presented as mean ± standard deviation (SD) of three independent experiments. Statistical analysis was performed using one-way ANOVA, followed by Dunnett’s post hoc test: **** *p* < 0.0001, *** *p* = 0.0002, * *p* = 0.0332, ns = not significant.

Cell viability was evaluated in HEK-293T and A72 cells following treatment with the CCoV-HRC peptide at concentrations ranging from 3 µM to 200 μM. After 24 h of exposure, dose–response curves were generated, and the cytotoxic concentration causing a 50% reduction in cell viability (CC_50_) was determined (Figure 1).

For HEK-293T, the CC_50_ was 192.7 µM (Figure 1A), whereas for A72 it was 167.5 µM (Figure 1B).

### 3.3. CCoV-HRC Peptide Inhibits CCoV-Mediated Cell Fusion

To evaluate the ability of CCoV-HRC to inhibit membrane fusion, we performed a cell–cell fusion assay based on β-gal complementation. HEK-293T cells expressing the APN receptor and the ω subunit of β-gal were mixed with cells expressing the CCoV S protein, the α subunit of β-gal, and the red fluorescent protein mCherry for fluorescence tracking.

Upon fusion between the two cell populations, the α and ω subunits of β-gal complement each other, generating a luminescent signal. Fusion was assessed in the presence or absence of the CCoV-HRC peptide, and luminescence was measured after 24 h.

**Figure 2:**
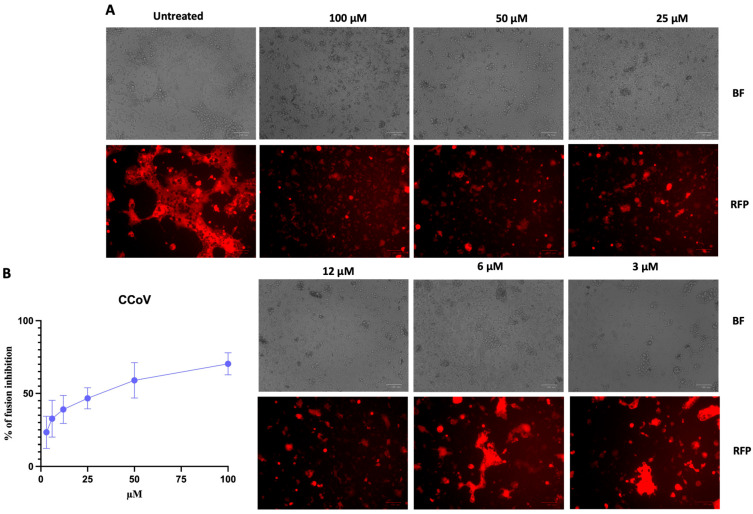
Inhibition of CCoV-mediated cell fusion in HEK-293T cells expressing the CCoV S protein and the APN receptor. (**A**) Representative images of the fusion assay acquired with the ZOE Fluorescent Cell Imaging System (Bio-Rad) in brightfield (BF) and red fluorescent protein (RFP) channels. Scale bar value = 100 μm. (**B**) Quantification of fusion inhibition based on β-gal complementation, measured using a Tecan M1000PRO microplate reader. Data are expressed as means (±standard deviation) from three independent experiments. Untreated cells (CTRL−), positive controls (CTRL+), and scrambled peptide (sequence: ENLLVLNNATLTFITEDNITIFLIRSENLNEHYLAENLTDVGLNKWID) were included for comparison. The scrambled peptide showed no significant inhibition of cell–cell fusion.

Figure 2 shows the representative images of the cell–cell fusion assays visualized by the mCherry signal.

In untreated samples, cells appeared fused and interconnected, forming extensive multinucleated networks. In contrast, in the presence of increasing concentrations of peptide, fusion was progressively inhibited, and cells appeared as isolated fluorescent spots. Quantification by luminescence confirmed a dose-dependent inhibition of fusion. At the highest concentration tested (100 μM), CCoV-HRC inhibited fusion by approximately 70%, and the half-maximal inhibitory concentration (IC_50_) was estimated to be 34 μM.

To determine whether the inhibitory activity of CCoV-HRC was specific for CCoV, we next evaluated its effect on SARS-CoV-2-mediated fusion. HEK-293T cells were transfected with the human ACE-2 receptor and the SARS-CoV-2 S protein, and cell–cell fusion was assessed using the same β-gal complementation assay described above.

**Figure 3:**
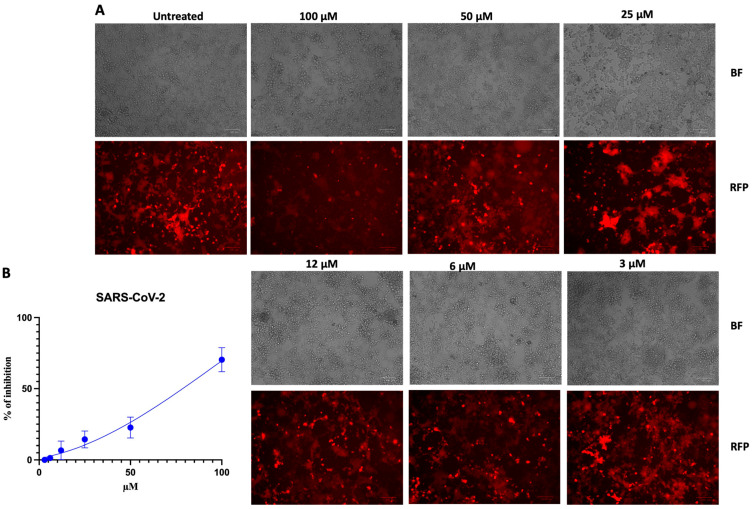
Inhibition of SARS-CoV-2-mediated cell fusion in HEK-293T cells expressing the SARS-CoV-2 S protein and the ACE2 receptor. (**A**) Representative images of the fusion assay acquired with ZOE Fluorescent microscopy (Bio-Rad) in bright-field (BF) and RFP channels. Scale bar value = 100 μm. (**B**) Quantification of fusion inhibition based on β-gal complementation by Tecan M1000PRO microplate reader. The values are means (±standard deviation) from three independent experiments.

As shown by the fluorescence images, the HRC-CCoV peptide inhibited S-mediated fusion only at the highest concentration tested (Figure 3).

Consistently, luminescence measurements revealed a markedly lower inhibitory potency against SARS-CoV-2, with an estimated IC_50_ of 79 µM. These results indicate that CCoV-HRC displays limited cross-reactivity toward SARS-CoV-2 and preferentially inhibits CCoV-mediated membrane fusion.

### 3.4. CCoV-HRC Exhibits Antiviral Activity Against Live CCoV

**Figure 4:**
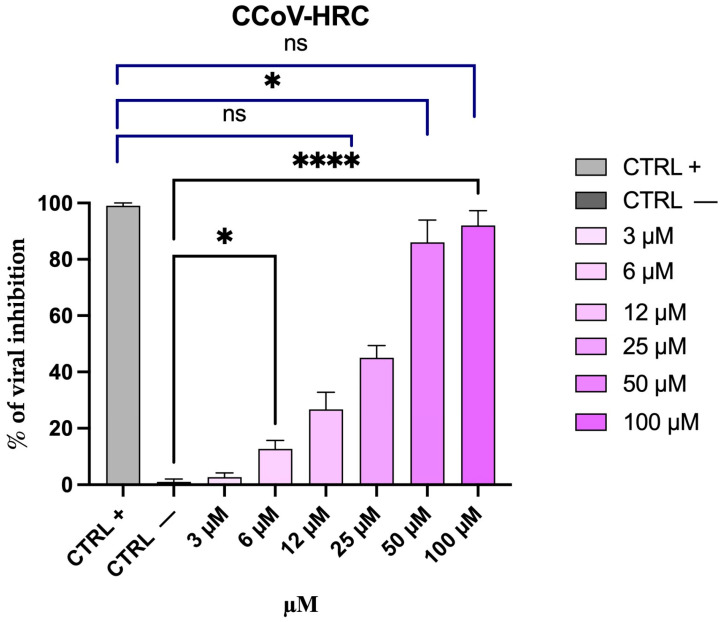
Antiviral activity of CCoV-HRC during in vitro CCoV infection in A72 cells. The % of viral inhibition was calculated using the TCID_50_ assay in virus pre-treatment experiments. Oreoch-1 peptide was used as a positive control (CTRL+) [23], whereas untreated and infected cells served as the negative control (CTRL−). The scrambled peptide (sequence: ENLLVLNNATLTFITEDNITIFLIRSENLNEHYLAENLTDVGLNKWID) was also added to the test, without reducing viral infectivity, confirming the sequence-specific activity of CCoV-HRC. Statistical analysis was performed by one-way ANOVA followed by Dunnett’s post hoc test. Significance compared to CTRL− (black lines) was **** *p* < 0.0001 and * *p* = 0.0332, while compared to CTRL+ (blue lines) was ns = 0.1234, * *p* = 0.0021, **** *p* < 0.0001.

The antiviral activity of CCoV-HRC was evaluated in vitro using CCoV type II, strain S/378. Viral particles were incubated with increasing concentrations of the peptide (3–100 µM) for 1 h at 37 °C and subsequently dispensed onto the A72 cell monolayer. After viral adsorption, the inoculum was replaced with fresh medium, and cells were incubated for 48 h. Viral inhibition was calculated using the TCID_50_ assay. CCoV-HRC significantly reduced viral load in a dose-dependent manner over the concentration range of 25–100 µM, with an IC50 of 25.6 µM (Figure 4). At the highest concentration tested (100 μM), 90% inhibition was observed.

These results demonstrate that CCoV-HRC effectively inhibits replication of live CCoV in vitro. Additionally, we calculated the peptide’s SI values for both cell lines and viruses. To clarify, these values are shown in Table 4 along with CC_50_ and IC_50_ data.

SI is a key parameter for assessing the therapeutic window of a compound. A high SI (≥10) indicates low toxicity and effective antiviral activity. In contrast, a low SI (<2) suggests considerable toxicity and poor selectivity [30,31]. CCoV-HRC has an SI of 5.6 and 6.5 in cell–cell fusion assays (HEK-293T) and in live virus (A72), respectively, indicating moderate selectivity and making it a promising candidate for optimisation. Conversely, for SARS-CoV-2, the SI is 2.4, reflecting low selectivity.

### 3.5. CCoV-HRC Downregulates the Expression of the Gene Encoding the S Protein

**Figure 5:**
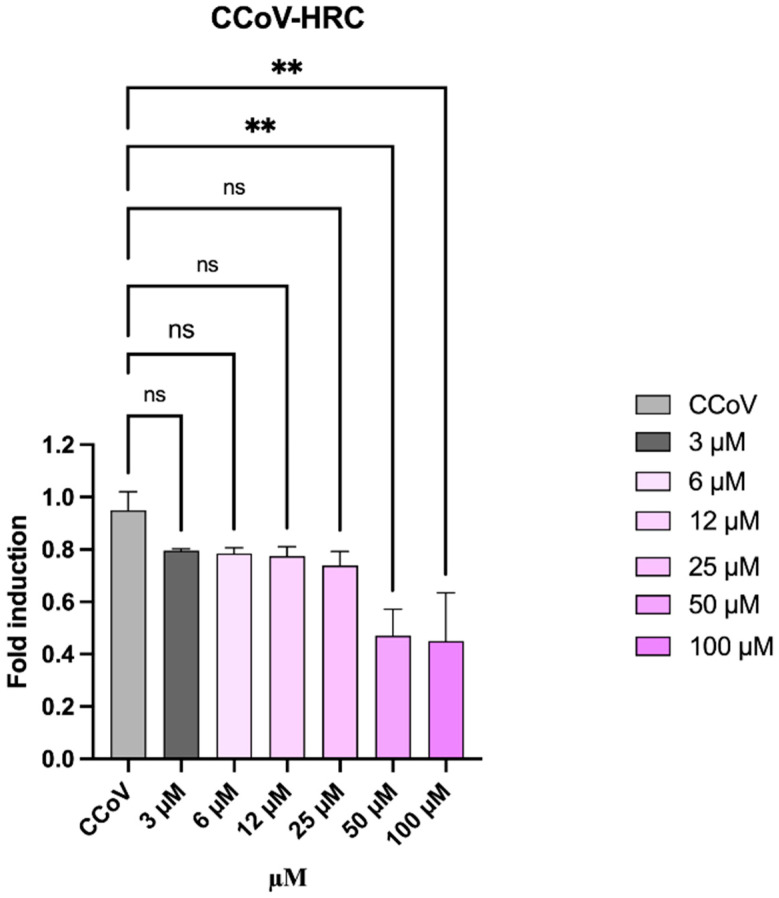
Quantitative real-time PCR analysis of CCoV *S* gene expression following virus pre-treatment with CCoV-HRC. One-way ANOVA followed by Dunnett’s multiple comparisons test was performed, ** *p* = 0.0021, ns = not significant.

The antiviral activity of CCoV-HRC was further confirmed at the molecular level during CCoV infection of A72 cells by qPCR. Following a virus pretreatment assay, total RNA was extracted, reverse-transcribed, and viral S gene expression was quantified. A significant reduction in S gene expression was observed at the highest peptide concentrations (Figure 5).

This data is consistent with the peptide interfering with early stages of viral fusion and entry, highlighting its ability to inhibit CCoV infection even when S protein levels are not substantially affected.

### 3.6. In Silico Modelling and Dynamic Analysis of Coronavirus Protein–Ligand Interactions

**Figure 6:**
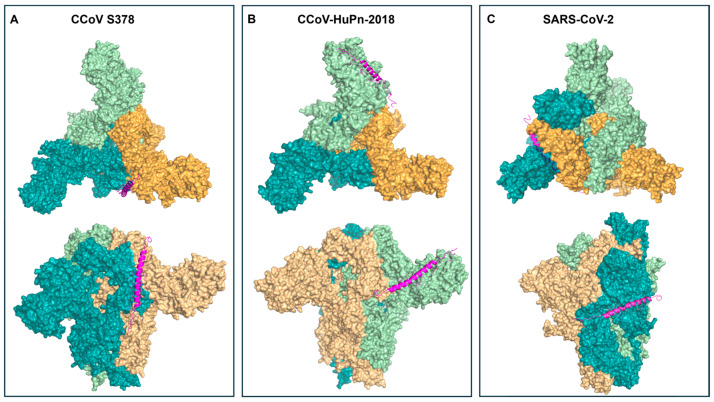
Docking results of the S protein of CCoV S378 (**A**), CCoV-HuPn-2018 (**B**), and SARS-CoV-2 (**C**) with the HRC peptide. The S protein chains of the trimer are shown in blue (chain A), green (chain B), and orange (chain C), while the CCoV-HRC peptide is represented as a magenta cartoon. (**A**) The HRC peptide binds to CCoV S378 between chain A (S2 subunit) and chain C (S1 subunit). (**B**) In HuPn-2018, the HRC peptide binds to the viral S1 subunit. (**C**) Finally, in SARS-CoV-2, the peptide binds to chain A between S1 and S2. Subunit identification was performed according to Tortorici et al. [32].

To investigate the molecular basis of ligand recognition by the S protein and fusion core of CCoV strain S378, CCoV-HuPn-2018, and SARS-CoV-2, an integrated in silico approach combining homology modelling, molecular docking, and MDS was employed. Due to the absence of a crystallographic structure for the CCoV S378 S protein in the PDB, a 3D model was first reconstructed through homology modelling (Figure 6).

After model validation (see Materials and Methods, Section 2.8), the spike proteins of all three viruses were docked with the CCoV-HRC peptide using LZerD. Despite sequence divergence (CCoV S378/HuPn-2018: 97.92% identity; CCoV S378/SARS-CoV-2: 31.67% identity), the overall 3D architecture was highly conserved. Structural inspection of the docked complexes revealed that the CCoV-HRC peptide binds within the interfacial region of the trimeric spike core, engaging residues from adjacent subunits.

In the lateral views, the peptide adopts an α-helical conformation and aligns parallel to the central helical axis of the fusion core. Its positioning is consistent with insertion into the hydrophobic groove formed by the HR1 helices of the trimer. Top-view representations further demonstrated that the peptide occupies the central channel of the trimeric assembly, establishing contacts with at least two neighboring spike chains simultaneously. In the CCoV S378 (Figure 6A) and CCoV-HuPn-2018 (Figure 6B) complexes, the peptide is deeply embedded within the inter-helical groove, spanning the interface between subunits and maintaining a longitudinal orientation along the HR1 bundle. This configuration supports a competitive binding mechanism in which the exogenous HRC peptide mimics the native HR2 region and sterically interferes with 6HB assembly.

In contrast, in the SARS-CoV-2 complex (Figure 6C), the peptide appears more peripherally positioned and less symmetrically distributed across the trimer interface. The interaction surface is reduced and does not fully occupy the central groove, suggesting weaker inter-subunit stabilization. Overall, these structural observations support a model in which effective inhibition of post-fusion core assembly requires stable multi-chain engagement of the HR1 trimeric groove, a condition achieved in CCoV S378 and CCoV-HuPn-2018 but only partially in SARS-CoV-2.

**Figure 7:**
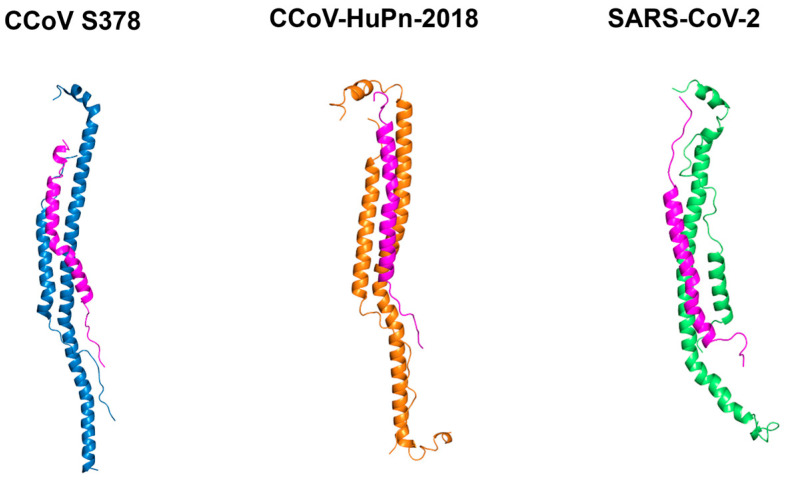
Individual structural models of the HR1–HR2 post-fusion cores from CCoV-S378, CCoV-HuPn-2018, and SARS-CoV-2, highlighting the CCoV-HRC peptide (magenta) bound antiparallel to the central HR1 coiled coil.

Similarly, the post-fusion cores of the three viruses were docked with the peptide to assess their potential interference with the viral fusion mechanism (Figure 7).

Individual structural models of the HR1–HR2 post-fusion cores from CCoV-S378, CCoV-HuPn-2018, and SARS-CoV-2 reveal a conserved trimeric coiled-coil architecture characteristic of class I viral fusion proteins. Overall, these structural models illustrate that CCoV-HRC adopts a canonical HR2-like binding mode, engaging the HR1 trimer in an anti-parallel configuration that is structurally compatible with competitive inhibition of 6HB assembly.

The resulting protein–peptide complexes were then subjected to MDS to characterize their dynamic behavior (Figure 7).

**Figure 8:**
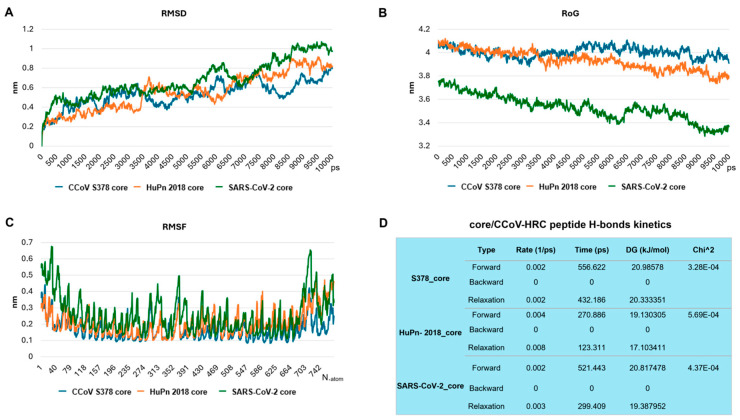
Stability and kinetic analysis of CoV core complexes. (**A**) RMSD and (**B**) RoG were calculated for the CCoV S378 core (blue), CCoV-HuPn-2018_core (orange), and SARS-CoV-2_core (green) complexes with the CCoV-HRC peptide over 10 ns of MDS. As shown in (**A**), the SARS-CoV-2 core complex reaches thermodynamic stability later compared to the other two complexes. RoG analysis (**B**) indicates that while the core conformation remained largely constant during the MD simulation in CCoV S378_and CCoV-HuPn-2018, it decreased in SARS-CoV-2, suggesting a more compact structure. (**C**) RMSF analysis of the three complexes shows the flexibility of individual atoms of the CCoV-HRC peptide. The X-axis represents the atom number of the HRC peptide, while the Y-axis represents atomic fluctuations in nanometers (nm). The results indicate that the HRC peptide exhibits greater flexibility—and thus lower stability—when bound to the SARS-CoV-2_core complex compared to the other cores. (**D**) H-bond kinetic analysis summary. This table provides a summary of the hydrogen bond dynamics for the three core complexes, including forward and backward rates, relaxation times, characteristic times, and the free energy changes (ΔG) associated with hydrogen bond formation and dissociation.

A preliminary 1 ns simulation was used to estimate binding energies, which revealed lower ΔG values for core/CCoV-HRC complexes compared to S protein/CCoV-HRC. Based on this, a longer 10 ns simulation was performed on the core/CCoV-HRC complexes to assess binding stability and dynamic interactions. RMSD analysis showed that all three complexes reached equilibrium within 10 ns; however, MDS of CCoV-HRC bound to the fusion cores revealed differential binding mechanisms across the three viral strains. After 10 ns, the HR1/HR2 helices remained in an extended, stable conformation in both CCoV S378 and CCoV-HuPn-2018 complexes, indicating effective structural stabilization by the peptide (Figure 8A). In contrast, the SARS-CoV-2 complex stabilized more slowly, exhibiting a conformational transition with HR1/HR2 helices beginning to fold, and suggesting reduced peptide-induced stabilization.

RoG analysis (Figure 8B) further highlighted differences in structural compactness: CCoV S378 and CCoV-HuPn-2018 maintained relatively constant RoG values, whereas SARS-CoV-2 displayed a pronounced decrease (from 3.75 nm to 3.35 nm), indicating peptide-induced conformational collapse rather than stabilization.

Finally, RMSF analysis (Figure 8C) revealed that while all complexes bound the peptide, the HRC peptide exhibited the largest fluctuations when bound to SARS-CoV-2. Average RMSF values were 0.2605 nm, 0.2608 nm, and 0.5004 nm for S378, HuPn-2018, and SARS-CoV-2, respectively.

Collectively, these MDS analyses provide mechanistic evidence that the HRC peptide effectively blocks the post-fusion complex in CCoV S378 and CCoV-HuPn-2018 by stabilizing and rigidifying the 6HB. In contrast, peptide binding to SARS-CoV-2 does not achieve equivalent inhibition, as indicated by slower stabilization, conformational rearrangement, and increased residue-level flexibility. Despite preserved hydrogen bonding across all three systems (Figure 8D), the results suggest that effective post-fusion blockade requires both sustained intermolecular contacts and structural rigidity—conditions fulfilled in CCoV S378 and HuPn-2018, but not in SARS-CoV-2.

## 4. Discussion

Membrane fusion is a crucial step for enveloped viruses to breach the host cell membrane and initiate the replication cycle [33]. For this reason, identifying molecules that act as viral entry inhibitors is particularly attractive, as such agents may be used as therapeutic or prophylactic tools to prevent infection.

HR regions are motifs located within the glycoproteins of enveloped viruses, assembling into a 6HB that drives the fusion between viral and cellular membranes [34,35,36,37]. HR-derived peptides have been widely recognized for their capacity to disrupt this process and effectively inhibit viral entry [38,39,40,41,42,43].

To date, no HR-based inhibitory peptides derived from the CCoV S protein have been reported. In this study, based on multiple sequence alignment of HR regions from different coronaviruses (Table 3), we designed a novel peptide, CCoV-HRC, derived from the C-terminal HR region of the CCoV S protein. Structural studies have recently defined the receptor-binding and fusion core regions of the strain CCoV–human pneumonia-2018 (CCoV-HuPn-2018), revealing a 23-turn helix for HR1 and a 9-turn helix for HR2, and demonstrating high entry efficiency via the APN receptor [32,44]. Therefore, we conducted a β-gal-based cell–cell fusion assay, demonstrating that CCoV-HRC effectively inhibits CCoV-mediated membrane fusion (Figure 2). This assay specifically models the interaction between the CCoV S protein and its cellular receptor APN, highlighting the ability of CCoV-HRC to interfere with the S-APN-driven fusion process. The peptide inhibited fusion with an IC_50_ of 34 µM, supporting a mechanism consistent with interference with the HR1–HR2 interaction within the S2 subunit. Based on the established fusion model of class I viral glycoproteins [14,15,16,17,18,19], it is plausible that CCoV-HRC interacts with the complementary HR1 region, thereby preventing the formation of the 6HB required for membrane merger. Importantly, the inhibitory activity observed in the fusion assay was confirmed in the context of live virus infection (Figure 4). In virus pre-treatment experiments using CCoV type II (strain S/378), CCoV-HRC significantly reduced viral replication in A72 cells in a dose-dependent manner, with an IC_50_ of 25.6 µM as determined by the TCID_50_ assay. These results demonstrate that interference with S-APN-mediated fusion translates into effective inhibition of CCoV infection, thereby validating the biological relevance of the fusion assay findings.

Molecular analysis by qPCR further supported these findings (Figure 5), showing a significant reduction in viral S gene expression following CCoV-HRC treatment. Together, the concordant results obtained from fusion assays, live virus inhibition, and transcriptional analysis provide strong evidence that CCoV-HRC acts as an entry inhibitor targeting the S2-mediated fusion step. Importantly, the peptide exhibited minimal cytotoxicity even at high concentrations (Figure 1), supporting its potential as a safe antiviral agent.

The peptide displayed a high degree of specificity, showing limited cross-inhibitory activity against heterologous coronaviruses. Indeed, in fusion assays against SARS-CoV-2 (Figure 3), CCoV-HRC exhibited a substantially higher IC_50_ (79 µM), indicating reduced potency. In the context of coronavirus fusion inhibitors, it is important to distinguish between virus-specific and pan-coronavirus strategies. Virus-specific inhibitors, such as CCoV-HRC, are designed to precisely complement the HR1 region of a single coronavirus species or closely related strains. This targeted interaction allows for high potency against the intended virus but typically limits cross-reactivity with other coronaviruses. In contrast, pan-coronavirus inhibitors, exemplified by EK1 and its derivatives, are engineered to engage conserved structural motifs within the HR1 grooves shared across multiple coronavirus genera [19,45,46]. Similarly, Xia et al. identified HR2-derived peptides from the bat coronavirus HKU4 that have activity against MERS-CoV [47,48]. By targeting broadly preserved HR interactions, these peptides achieve cross-species inhibitory activity, although often with slightly lower potency compared to virus-specific peptides.

Evidence from animal CoV further supports the relevance of HR-derived peptides as effective fusion inhibitors. HR2-derived peptides have been successfully developed against PEDV, where Zhao et al. identified three peptides (HR2M, HR2L, and HR2P) as potent antivirals, with HR2P showing the strongest inhibition and eliciting neutralizing antibodies in mice [49]. Similarly, Liu et al. described several fusion-inhibitory peptides derived from the S protein of FCoV [50]. Notably, the HRC-derived FP4 peptide displayed enhanced antiviral activity due to the presence of NNTLVNL residues at its C-terminus, which were suggested to play a critical role in membrane fusion inhibition. Interestingly, these same residues are conserved in the CCoV-HRC sequence designed in this study, suggesting a shared structural and functional role among animal alphacoronaviruses. Currently, there are no specific treatments for canine coronavirus. Liu et al. reported the activity of a derivative of Remdesivir VV116 against human and animal coronaviruses, including CCoV [51]. Ho et al. found that GC376 effectively inhibited the protein activity of CCoV Mpro at sub-molar concentrations [52]. Therefore, these compounds act on viral replication after the virus binds to and enters the host cell. Interestingly, Tortorici et al. reported that human plasma-derived antibodies following HCoV-229E infection inhibited spike protein-mediated entry of CCoV-HuPn-2018, indicating the presence of cross-reactive antibodies between alphacoronaviruses [32]. In that study, they also employed an anti-TGEV/PRCV monoclonal antibody targeting the APN receptor domain, which blocks the S protein from attaching to the receptor before the fusion event.

Investigating peptide inhibitory activity across diverse coronavirus models is critical for identifying optimized lead compounds tailored to specific viral targets. While developing a broad-spectrum pan-coronavirus inhibitor is a viable long-term goal, focusing on the unique molecular signatures of individual viruses remains the most effective strategy for maximizing inhibitory efficacy, providing the best foundation for developing the most effective clinical treatments. A virus-specific approach is able to ensure potent therapeutic intervention strategies to be prepared for future outbreaks with specialized therapeutics that offer higher precision and specificity compared to broad-spectrum alternatives.

Our in silico analyses further support the biological data (Figure 6, Figure 7 and Figure 8), revealing that CCoV-HRC stably engages the HR1 groove and mimics the native HR2 region to prevent 6HB formation. Docking (Figure 6 and Figure 7) and MSD (Figure 8) demonstrated strong multi-chain interactions in CCoV S378 and CCoV-HuPn-2018, whereas reduced binding stability and increased flexibility were observed with SARS-CoV-2, consistent with the lower inhibitory activity (Figure 3). These findings provide a structural rationale for the virus-specific efficacy of the peptide and highlight the utility of computational modeling in guiding peptide-based antiviral design.

Taken together, our results indicate that CCoV-HRC represents a promising candidate for the treatment and prevention of CCoV infections, for which no specific approved antiviral agents are currently available. However, the present study is limited to in vitro analyses. Future studies employing appropriate animal models will be essential to evaluate in vivo efficacy, pharmacokinetics, stability, and safety, which represent common challenges in peptide antiviral development. Indeed, an important consideration for peptide-based antivirals is their stability and delivery. Peptides can be susceptible to proteolytic degradation and may exhibit limited bioavailability in vivo, which can reduce therapeutic efficacy. Strategies to overcome these challenges include chemical modifications such as N-terminal acetylation [53,54], C-terminal amidation [46,55], cyclization [56,57], or lipid/cholesterol conjugation [58], which can enhance peptide stability, membrane permeability, and half-life. Additionally, formulation approaches, such as encapsulation in nanoparticles or delivery via inhalation for respiratory viruses [59,60], may improve tissue targeting and pharmacokinetics. While our study focused on in vitro activity, these considerations will be critical in future preclinical evaluations to translate CCoV-HRC into an effective antiviral therapeutic.

Although the functional data strongly support an entry-inhibitory mechanism, the present study does not include direct biophysical characterization of the interaction between CCoV-HRC and the HR1 region of the spike protein. Quantitative binding assays such as surface plasmon resonance (SPR), biolayer interferometry (BLI), microscale thermophoresis (MST), isothermal titration calorimetry (ITC), or interaction studies using recombinant HR1-derived peptides or stabilized fusion-core constructs would provide definitive evidence allowing determination of binding affinity and specificity. Finally, another limitation of this study is that the CCoV-HRC peptide was tested only against a single CCoV genotype, and its activity against other genotypes or point mutations in the HR region remains to be evaluated.

## Figures and Tables

**Table 1:** Viral protein sequences and crystallographic structures retrieved from NCBI and PDB databases.

Virus Strain	Protein (Region)	NCBI Accession	PDB Accession
CCoV S378	Spike (full-length)	KC175341	Modelled
CCoV-HuPn-2018	Spike (full-length)	QVL91811	7US6
SARS-CoV-2	Spike (full-length)	QHD43416.1	7QUS
CCoV S378	Fusion core (HR1–HR2)	Derived from KC175341	Modelled
CCoV-HuPn-2018	Fusion core (HR1–HR2)	MW591993	8X7Z
SARS-CoV-2	Fusion core (HR1–HR2)	Derived from NC_045512	7COT

**Table 2:** Bioinformatics tools used in this study.

Tool	Purpose	Version/Access
SWISS-MODEL	Homology modelling	https://swissmodel.expasy.org/ (accessed on 27 February 2026)
LZerD	Protein–peptide docking	https://lzerd.kiharalab.org/ (accessed on 26 February 2026)
Chiron	Structure refinement	https://dokhlab.med.psu.edu/chiron (accessed on 26 February 2026)
ProCheck	Stereochemical validation	v.3.5.4
GROMACS	Molecular dynamics simulations	v.2023.2
PyMOL	Structural visualization	v.2.5.8

**Table 3:** HRC sequences of different CoVs.

Genus	Peptide	Sequence	Identity	Similarity
α-CoVs	CCoV-HuPn 2018	DIFNATYLNLTGEIDDLEFRSEKLHNTTVELAILIDNINNTLVNLEWL	97.92%	100%
	TGEV	DIFNATYLNLTGEIDDLEFRSEKLHNTTVGLAILIDNINNTLVNLEWL	95.83%	97.92%
	PEDV	DVFNATYLKLTGEIADLEQRSESLRNTTEELQSLIYNINNTLVDLEWL	77.08%	81.25%
	FCoV	EIFNQTKLNLTAEIDQLEQRADNLSTIAHELQQYIDNLNNTLVDLEWL	58.82%	72.55%
	FIPV	DIFNQTKLNLTAEIDQLEQRADNLTTIAHELQQYIDNLNKTLVDLDWL	58.82%	70.59%
β-CoVs	SARS-CoV	GIINNTVYDPLQPELDSFKEELDKYFKNHTSPDVDLGDISGINASVVNIQKE	34.48%	48.28%
	SARS-CoV-2	DVDLGDISGINASVVNIQKEIDRLNEVAKNLNESLIDLQELGKYE	31.67%	45%
	MHV	DKWFKNQTSIAPDLSLDFEKLNVTFLDLTYEMNRIQDAIKKLNESYINLKE	30.51%	50.85%
γ-CoVs	IBV	DFNYTVPILNISGEIDNIQGVIQGLNDSLINLEELSIIKTYIKWPWYVWL	28.12%	42.19%

CCoV-HuPn 2018 (Canine coronavirus HuPn-2018); TGEV (Transmissible gastroenteritis virus); PEDV (Porcine epidemic diarrhea virus); MHV (mouse hepatitis virus); FCoV (Feline coronavirus); FIPV (Feline Infectious Peritonitis Virus); SARS-CoV (Severe Acute Respiratory Syndrome Coronavirus); SARS-CoV-2 (Severe Acute Respiratory Syndrome Coronavirus 2); IBV (Infectious Bronchitis Virus); MHV (mouse hepatitis virus). Identity = percentage of identical amino acids at the same positions between two aligned sequences. Similarity = includes identity and amino acid substitutions that maintain chemical–physical properties.

**Table 4:** CC_50_, IC_50,_ and SI values of CCoV-HRC peptide against CCoV and SARS-CoV-2.

CCoV-HRC Peptide (µM)						
	CCoV			SARS-CoV-2		
	CC_50_	IC_50_	SI	CC_50_	IC_50_	SI
HEK-293T	192.7	34	5.6	192.7	79	2.4
A72	167.5	25.6	6.5	-	-	-

## Data Availability

The original contributions presented in this study are included in the article. Further inquiries can be directed to the corresponding author.

## Abbreviations

The following abbreviations are used in this manuscript:

CCoV	Canine coronavirus
CoVs	Coronaviruses
FCoV	Feline Coronavirus
APN	aminopeptidase N
FP	fusion peptide
HR	heptad repeat
HR1	N-terminal HR domain
HR2	C-terminal HR region
6HB	six-helix bundle
HIV-1	human immunodeficiency virus type 1
FDA	Food and Drug Administration
SARS-CoV	severe acute respiratory syndrome coronavirus
SARS-CoV-2	severe acute respiratory syndrome coronavirus type 2
MERS-CoV	Middle East respiratory syndrome
FIPV	feline infectious peritonitis virus
MTT	3-(4,5-dimethyl-2-thiazolyl)-2,5-diphenyl-2H-tetrazolium bromide
DMSO	dimethyl sulfoxide
CPEs	cytopathic effects
TCID_50_	tissue culture infective dose
qPCR	real-time quantitative PCR
Ct	Cycle threshold
GAPDH	glyceraldehyde-3-phosphate dehydrogenase
IBV	avian infectious bronchitis virus
TGEV	transmissible gastroenteritis coronavirus
MHV	mouse hepatitis virus
PEDV	porcine epidemic diarrhea virus
